# Amperometric Cytosensor for Studying Mitochondrial Interferences Induced by Plasticizers Bisphenol B and Bisphenol A

**DOI:** 10.3390/molecules25215185

**Published:** 2020-11-07

**Authors:** Roberto Dragone, Gerardo Grasso, Chiara Frazzoli

**Affiliations:** 1 Istituto per lo Studio dei Materiali Nanostrutturati, Consiglio Nazionale delle Ricerche, c/o Dipartimento di Chimica, ‘Sapienza’ Università di Roma, Piazzale Aldo Moro, 5, 00185 Rome, Italy; roberto.dragone@cnr.it; 2 Dipartimento Malattie Cardiovascolari, Dismetaboliche e dell’Invecchiamento, Istituto Superiore di Sanità, Via Giano della Bella, 34, 00162 Rome, Italy; chiara.frazzoli@iss.it

**Keywords:** food contact materials, toxicology, yeast, mitochondria, uncouplers, oxidative stress

## Abstract

The widespread presence of plasticizers Bisphenol B (BPB) and Bisphenol A (BPA) in food contact materials, medical equipment, and common household products is a toxicological risk factor for health due to internal exposure after environmental dietary exposure. This work describes the use of an amperometric cytosensor (i.e., a whole cell-based amperometric biosensoristic device) for studying mitochondrial interferences of BPA and BPB (5–100 µg/mL) in the yeast *Saccharomyces cerevisiae* model following long-term (24 h) exposure (acute toxicity). Percentage interference (%ρ) on yeast aerobic mitochondrial catabolism was calculated after comparison of aerobic respiration of exposed and control *S. cerevisiae* cell suspensions. Results suggested the hypothesis of a dose-dependent co-action of two mechanisms, namely uncoupling of oxidative phosphorylation and oxidative stress. These mechanisms respectively matched with opposite effects of hyperstimulation and inhibition of cellular respiration. While uncoupling of oxidative phosphorylation and oxidative stress have been previously described as separate effects from in vitro BPA exposure using other biochemical endpoints and biological systems, effects of BPB on cellular aerobic respiration are here reported for the first time. Results highlighted a similar hyperstimulation effect after exposure to 5 µg/mL BPA and BPB. About a 2-fold higher cellular respiration inhibition potency was observed after exposures to 15, 30, and 100 µg/mL BPB compared to BPA. 2,4-Dinitrophenol (2,4-DNP) was used as model uncoupling agent. A time-dependent mechanism of mitochondrial interference was also highlighted.

## 1. Introduction

Bisphenol A (BPA; 2,2-bis(4-hydroxyphenyl)propane) is an industrial plasticizer widely used for the production of epoxy resins, phenol resins, polycarbonates, polyacrylates, and polyesters. It finds application in the manufacture of many types of products, including food containers (e.g., baby bottles and water carboys) and food contact materials, thermal paper, and medical devices (e.g., respirators). Biomonitoring data suggested that, notwithstanding its short biological half-life [1,2,3], BPA is consistently found in human biological samples, including umbilical cord blood and colostrum, as a result of widespread, continuous and/or repeated exposure. BPA is a well-known estrogen-like endocrine-disrupting chemical [4,5]. Besides transgenerational exposure [6] and sustainable food safety aspects [7], bottle-fed infants are a population group most exposed to dietary BPA [8,9]. The increasing worldwide banning or restrictions on BPA-containing baby bottles due to safety concerns could worsen the scenario of infant exposure in countries that are dumping destination [10,11]. BPA and BPA analogues can enter the food supply chain at various stages [12,13]. Further research is needed to fill the gaps and uncertainties on the toxicological profile of BPA and its proximate substitutes. Indeed, BPA has been extensively studied mainly for its endocrine disrupting activity but not for other possible harmful effects like mitochondrial interference. Concerning BPA substitutes such as BPF, BPS, BPAF, BPC, BPP, and BPB, toxicological profiles are even more incomplete. Bisphenol B (BPB; 2,2-Bis(4-hydroxyphenyl)butane) is used as a monomer alternative to BPA in the manufacturing of various materials (e.g., polycarbonate plastic and resin) in commercial products [14]. BPB is currently used as a BPA alternative in countries such as the United States, where it has been registered by the Food and Drug Administration as an indirect additive used in certain food-contact coatings and polymers [15]. This bisphenol is relatively more resistant to biodegradation than BPA in aquatic environments [16]; it is a potential food contaminant, e.g., of canned foods and milk [17,18,19]. BPB has been found in human blood serum and urine [20,21] as well as in indoor dust [22]. BPB exerts estrogenic effects similar to BPA [23,24,25,26,27], but its toxicological profile is still more incomplete. Mitochondria are cell organelles that play a key role in cell bioenergetics and ATP synthesis through cellular aerobic respiration. Mitochondrial dysfunctions, including alterations of cellular redox homeostasis and cell metabolism, have been associated with processes like aging, the insurgence of neurodegenerative diseases, and carcinogenesis [28]. Impairments of mitochondrial functions have been reported by in vitro studies on cultured cells and isolated mitochondria exposed to BPA 1–100 µg/mL. Mitochondrial dysfunctions include uncoupling of oxidative phosphorylation and partial inhibition of mitochondrial electron transport chain complexes [29], reactive oxygen species (ROS) overproduction [30], changes in mitochondrial enzymes activities, ATP content, mitochondria ultrastructure, mitochondrial membrane permeability, mitochondrial membrane potential and lipid peroxidation levels [31]; alteration of the glutathione synthesis and recycle [32], and decrease of bovine spermatozoa motility (closely related to mitochondrial activity) [33]. As reported in a recent in vitro study conducted on human peripheral blood cells, the exposure to a BPB concentration range 200–800 µM (about 50 µg/mL–200 µg/mL) increased ROS and lipid peroxidation levels, and decreased GSH [34]. To date, no effects on cellular respiration have been reported in the literature for in vitro exposure to BPB. The cellular aerobic respiration is related to cellular O_2_ consumption, and the final steps of aerobic cellular respiration occur in mitochondria. The cellular aerobic respiration has already proved to be a valuable biochemical and toxicological endpoint in our previous studies on mitochondrial effects induced by toxicants [35,36,37,38,39]. Such studies were carried out using the model yeast *Saccharomyces cerevisiae* as mediator in electrochemical biosensoristic devices. Electrochemical biosensoristic devices have interesting analytical features that can be exploited for rapid and easy-to-use in vitro bioassays that are useful for both toxicological studies and automated field monitoring and diagnostics [40,41]. Whole cell-based biosensoristic devices (referred in this work as ‘cytosensors’, see Section 4.2) are characterized by a peculiar analytical approach. Through the selection of a given cellular function and an appropriate in vitro biochemical and toxicological endpoint, cytosensors can provide useful information about the bioactivity of the tested chemical against the selected cellular function [37,38]. In the present study, an amperometric cytosensor was used in respirometric bioassays to study the mitochondrial interferences of BPA and BPB in the model yeast *Saccharomyces cerevisiae* assessing acute toxicity following long-term (24 h) exposure. The adverse effects of acute toxicity can usually occur within 24 h of exposure to the toxicant. Among BPA proximate substitutes, BPB was chosen because its molecular structure is closely related to that of BPA, i.e., an additional methyl group is present in the hydrocarbon bridge of BPB molecule. The organic compound 2,4-Dinitrophenol (2,4-DNP) was used as a model chemical for its known mechanism of alteration of mitochondrial functions (uncoupling of oxidative phosphorylation) [42] in *S. cerevisiae* [43].

## 2. Results

**Figure 1:**
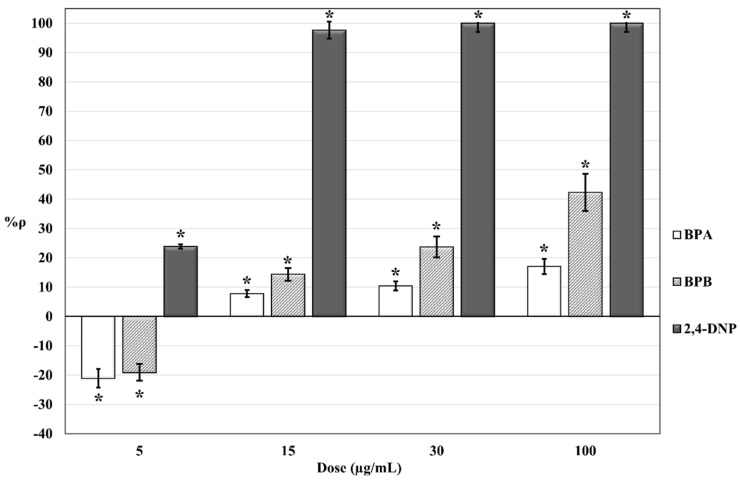
Dose-effect histogram of 24 h exposure of *S. cerevisiae* cell suspensions to BPA, BPB, and 2,4-DNP. %RSD ≤ 15%; ANOVA testing using randomized block design was applied for statistical differences between means values of Δppm O_2 exp_ and Δppm O_2 blk_. Statistical significance of the results is indicated with asterisks (* *p* ≤ 0.05).

The results of the respirometric bioassays on *S. cerevisiae* cell suspensions exposed to different concentrations of BPA, BPB, and 2,4-DNP are summarized in Figure 1.

Statistical tests were done using ANOVA testing for randomized bock design. A significant relationship between the mean variation of dissolved O_2_ in exposed and control *S. cerevisiae* cell suspensions (Δppm O_2 exp_ and Δppm O_2 blk_, see Section 4.2) was found at all concentrations tested (*p* ≤ 0.05). The use of randomized block design of ANOVA testing helped to reduce possible confounding factors that may affect the comparison between data acquired in different days of testing, e.g., possible differences between stock yeast cell suspensions prepared on different days.

**Figure 2:**
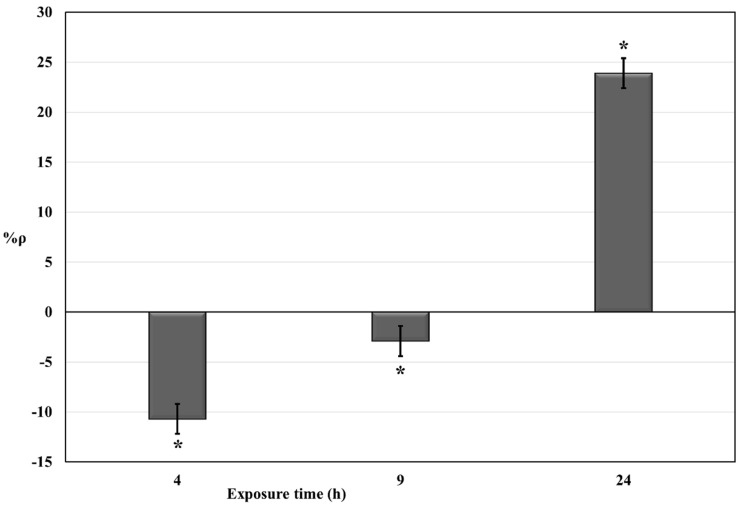
Dose-effect histogram of 5 µg/mL 2,4-DNP exposure (exposure times ≤ 24 h) of *S. cerevisiae* cell suspensions. %RSD ≤ 15%; ANOVA testing using randomized block design was applied for statistical differences between means values of Δppm O_2 exp_ and Δppm O_2 blk_. Statistical significance of the results is indicated with asterisks (* *p* ≤ 0.05).

Negative values of %ρ are expected to indicate an increase in cellular O_2_ consumption in the exposed *S. cerevisiae* cell suspensions (compared to control cell suspensions) due to hyperstimulation of mitochondrial activity. Positive values are expected to indicate a decrease in cellular O_2_ consumption in the exposed *S. cerevisiae* cell suspensions (compared to control cell suspensions) related to inhibition of mitochondrial activity. Long-term exposure of *S. cerevisiae* cells to increasing doses (from 5 to 100 µg/mL) of bisphenols determined an increase of %ρ from negative values, i.e., hyperstimulatory effect dominates (%ρ = −21,04% and %ρ = −19.05% for exposure to 5 µg/mL BPA and 5 µg/mL BPB, respectively), to positive values (for bisphenol concentrations ≥ 15 µg/mL), i.e., inhibitory effect dominates. Same doses of 2,4-DNP shown a higher toxic potency (compared to bisphenols). Short-term (4 h and 9 h) exposures to 5 µg/mL 2,4-DNP were performed (Figure 2).

## 3. Discussion

The dose-effect relationship for long-term (24 h) exposure to increasing doses (5–100 µg/mL) of BPA, BPB, and 2,4-DNP (Figure 1) shows how at same administered doses, differences in %ρ values for BPA, BPB, and 2,4-DNP can be ascribed to differences in toxic potencies. Such trends in %ρ values could be attributable to a dose-dependent co-action and combined effects of two presumably sequential mechanisms on cellular aerobic respiration, i.e., uncoupling of oxidative phosphorylation and oxidative stress. These mechanisms fit well with measured opposite effects of hyperstimulation of cellular aerobic respiration (negative %ρ values) and inhibition of cellular respiration (positive %ρ values). These findings are corroborated by a previously reported effect of BPA (1–200 µg/mL) on bovine spermatozoa motility (closely related to mitochondrial activity) [33]. Concerning hyperstimulation of cellular aerobic respiration, preliminary tests have proven that in our experimental conditions, *S. cerevisiae* cells are in an optimal physiological state for cellular aerobic respiration, i.e., maximal respiration rate. The observed hyperstimulation of *S. cerevisiae* cells exposed to BPA and BPB is expected to be the consequence of a partial uncoupling of oxidative phosphorylation in mitochondria, as previously reported in the literature for isolated rat hepatocytes mitochondria exposed to similar concentrations of BPA and other bisphenols [29].

Positive %ρ values in Figure 1 can rather be associated with the inhibition of cellular respiration in exposed *S. cerevisiae* cells. This effect is expected to be the consequence of oxidative stress caused by a ROS overproduction. Indeed, ROS overproduction may lead to irreversible damages of membrane lipids and proteins, resulting in detrimental effects for mitochondrial structures and functions, including aerobic respiration. Bereketoglu et al. (2017) have reported the expression of genes associated with the response to oxidative stress in *S. cerevisiae* exposed to BPA [44]. Oxidative stress and ROS overproduction induced by exposures to BPA concentrations higher than 5 µg/mL have been previously reported in the literature using different biological and toxicological endpoints and biological model systems [29,30,31,32,33]. To explain ROS overproduction in exposed *S. cerevisiae* cells, a parallel or sequential involvement of hyperstimulation of cellular respiration and electron flow slowdown through the mitochondrial electron transport chain complexes can be hypothesized. Under normal conditions, O_2_ is reduced to H_2_O by cytochrome c oxidase (Complex IV), and the release of the intermediate compounds of partially reduced O_2_ is very low. As a result of the uncoupling of oxidative phosphorylation, the simultaneous increase of O_2_ consumption could promote an incomplete O_2_ reduction and ROS overproduction at the level of Complex IV. Most studies report an inverse correlation between the uncoupling of oxidative phosphorylation and ROS production [45,46,47]. However, many of these studies were performed on isolated mitochondria. Some evidence suggests that a partial mitochondrial uncoupling of oxidative phosphorylation in whole cells could increase the level of detectable ROS [48]. Therefore, in our experimental conditions, it is reasonable to assume a direct correlation between uncoupling and ROS overproduction. The electron flow slowdown through the mitochondrial electron transport chain complexes (caused by a partial inhibition of Complex II) could also be hypothesized as mechanisms involved in ROS overproduction in exposed *S. cerevisiae* cells. Results reported by Nakagawa and Tayama show that the exposure of isolated rat hepatocyte mitochondria to similar concentrations of bisphenols can partially inhibit the mitochondrial electron transport chain of Complex I and Complex II, i.e., NAD**^+^**- and FAD-linked respiration [29]. A slight inhibition of the Complex IV has also been reported by Nakagawa and Tayama, but values for treated mitochondria were not significantly different from values for untreated mitochondria [29]. Contrary to mammals, *S. cerevisiae* mitochondria do not possess a multi-subunit Complex I-type NADH dehydrogenase [49]. Therefore, a partial inhibition of Complex II in *S. cerevisiae* exposed to BPA and BPB is plausible.

Our results show that BPB possesses a similar or even higher toxic potency for mitochondrial interference compared with BPA. Whereas at 5 µg/mL exposure concentration, a similar hyperstimulation effect was observed for both bisphenols (%ρ _BPB_ = −19.05% and %ρ _BPA_ = −21.04%), the results for exposure concentrations from 15 to 100 µg/mL showed a 2-fold higher cellular respiration inhibition potency for BPB compared to BPA (see Figure 1). This difference in toxic potency for mitochondrial interference could be ascribed to differences in structure and hydrophobicity between bisphenols. Nakagawa and Tayama [29] suggested a direct correlation between the toxic potency of bisphenols and the relative length/molecular weight of the hydrocarbon bridge between the two phenyl groups in bisphenols. Differences between cytotoxic effects observed in rat hepatocytes exposed to different bisphenols might be dependent on a different affinity of bisphenol molecules for the lipid core of biological membranes [29]. The interaction of BPA and other bisphenols with phosphatidylcholine and cardiolipin has been previously described [50,51,52,53]. Both phosphatidylcholine and cardiolipin are two of the major phospholipid constituents of mitochondrial membranes of eukaryotic cells (including *S. cerevisiae*) [54,55,56]. More specifically, the hydrocarbon bridge of bisphenols would appear to be directly involved in the interactions with phospholipids [51,52], and the strength of the interactions appears to be mainly linked to the hydrophobicity of the bisphenols [53]. Concerning our study, the presence of an additional methyl group in the hydrocarbon bridge of BPB molecule (compared to BPA) and the higher hydrophobicity of BPB (log Kow BPB = 4.13, log Kow BPA = 3.32) could therefore explain the higher inhibition potency of aerobic cellular respiration observed in *S. cerevisiae*. It can be hypothesized that there is a role for BPA and BPB interactions with mitochondrial phospholipids phosphatidylcholine and cardiolipin in bioconcentration and accumulation of bisphenols at a mitochondrial level, as well as in the resulting interferences on mitochondrial bioenergetics. In particular, cardiolipin plays a key role in several reactions and processes involved in mitochondrial bioenergetics, e.g., for the optimal enzymatic activity of oxidative phosphorylation complexes [57]. However, further studies are necessary to support this hypothesis.

2,4-DNP was used as a reference compound for the uncoupling of oxidative phosphorylation in *S. cerevisiae* [43]. In our experimental conditions, no hyperstimulation of cellular aerobic respiration was observed for 24 h exposure to 2,4-DNP (Figure 1). A set of respirometric bioassays for short-time exposures (4 h and 9 h) to 5 µg/mL 2,4-DNP were carried out. The dose of 5 µg/mL was chosen because at this concentration the hyperstimulation effect was observed for bisphenols after 24 h-exposure (Figure 1). The results obtained (Figure 2) confirmed a lower toxic potency for bisphenols in comparison with that of 2,4-DNP. This is possibly attributable to different toxicodynamics and/or kinetics of the trigger and action of mechanisms involved. In addition to a dose-dependent resultant effect of mitochondrial interference observed in *S. cerevisiae* cells for 24 h exposure time (Figure 1), these results also suggested a presumptive time-dependence resultant effect of mitochondrial interference for a fixed dose of 5 µg/mL (Figure 2). In both cases, the resultant effect of mitochondrial interference is mainly characterized by a gradual increase in inhibition of cellular respiration that may occur together or sequentially with respect for hyperstimulation. Further studies will help to elucidate the time-dependence features of the resultant effect of mitochondrial interference observed.

## 4. Materials and Methods

### 4.1. Solutions

All 100 µg/mL stock solutions were prepared in high-purity deionized water (Milli-Q system, Merck Millipore, Billerica, MA, USA) for all chemicals tested BPA (≥99% pure Sigma Aldrich, Milan, Italy), BPB ≥98.0% pure (TCI Europe nv, Antwerp, Belgium) and 2,4-DNP 98% pure (ACROS Organics, Fairrun, NJ, USA). Stock solutions were kept dark and stored at 4 °C in glass bottles. Before each respirometric bioassays, spectrophotometric measurements against high-purity deionized water were carried out at 25 °C on 1/20 dilutions of 100 µg/mL stock solutions of BPA (λ_max_ = 275 nm; 0.069 ± 0.004 absorbance units or AU), BPB (λ_max_ = 276 nm; 0.065 ± 0.003 AU) and 2,4-DNP (λ_max_ = 355 nm; 0.277 ± 0.006 AU) (Unicam UV2 UV/Visible spectrophotometer, Unicam Instruments Ltd, Arbury Road, Cambridge, United Kingdom). All measurements were carried out in quartz cuvettes with 1 cm optical path lengths. A 3 mol/L glucose aqueous stock solution was weekly prepared from glucose D(+) 99.5% GC (Sigma Aldrich, Milan, Italy). Between different bioassays, the glucose stock solution was stored in a sterilized glass bottle at 4 °C. For all the respirometric bioassays, the final concentration of 0.5 mol/L glucose was used as a metabolic substrate for *S. cerevisiae* cells. Preliminary respirometric bioassays have allowed us to assess the 0.5 mol/L glucose concentration as optimal for the achievement of the maximum rate of cellular respiration (excess metabolic substrate). The 0.02 mol/L stock solution of sodium azide, NaN_3_ (≥99.0%, AMS Biotechnology Ltd., Massagno, Switzerland) was prepared in high-purity deionized water and stored at 4 °C. For the calibration of Clark-type oximeters, a 10 g/L sodium sulfite solution (Na_2_SO_3_ ≥98% Sigma Aldrich, Milan, Italy) was prepared daily in high-purity deionized water.

### 4.2. Assembly of Amperometric Cytosensors and Respirometric Bioassay Set-Up

The amperometric cytosensor is made up of two main components: (i) a yeast cell suspension as biomediator, and (ii) a Clark-type oximeter (model 360, Amel Instruments S.r.l.; Milan, Italy) as the amperometric transducer measuring changes in dissolved oxygen (O_2_) concentration in the yeast cell suspension. Before the assembly of the amperometric cytosensors, a two-point calibration of the Clark-type oximeters was performed at 25 °C by measuring O_2_ in open air and dissolved O_2_ in a sodium sulfite solution (10 g/L). The amount of atmospheric O_2_ depends on both temperature and barometric pressure [39]. The value of 8.35 ppm O_2_ in air at 25 °C is reported in a table provided by manufacturers of Clark-type oximeters (Amel S.r.l). The calibration point with sodium sulfite gives a zero-reference point since this sodium sulfite reacts with the dissolved O_2_ to form sulfate. Stock yeast cell suspensions (500 mg/mL) were prepared daily, starting from 50.0 mg ± 0.1 mg of commercially active dried baker’s yeast (Mastro Fornaio PaneAngeli Cameo S.p.a., Desenzano del Garda (BS), Italy), rehydrated overnight with 10 mL of Milli-Q water in sterilized test tubes. Grasso et al. [36] previously defined the most suitable cell concentration of the stock yeast cell suspension (1.5 × 10^8^ colony forming units/mL or cfu/mL) as well as the 1:100 dilution ratio of the stock yeast cell suspension for the assembly of the amperometric cytosensors.

For each exposure test and respirometric bioassay, a set of eight amperometric cytosensors was used. The experimental set-up included four blank yeast cell suspensions (as control) and four exposed yeast cell suspensions:

- Blank yeast cell suspension: 12.50 mL of high-purity deionized water + 0.15 mL of 500 mg/mL of stock yeast cell suspensions
- Exposed yeast cell suspension: 12.50 mL of BPA, BPB, or 2,4-DNP solution + 0.15 mL of 500 mg/mL of stock yeast cell suspensions

**Figure 3:**
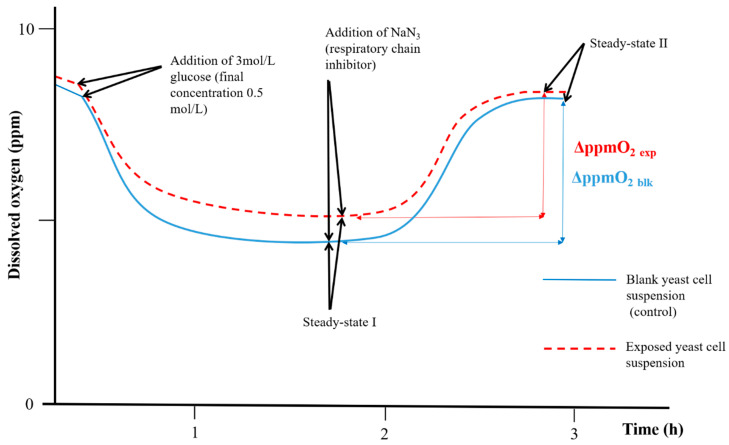
The experimental respirometric curve shows the dissolved oxygen (ppm O_2_) vs time (adapted from Dragone et al. [35]).

The same stock yeast cell suspension was used to prepare both blank yeast cell suspension control group and exposed yeast cell suspension group. Thus, any confounding variables would be identical for both groups. Throughout the 24 h exposures and respirometric bioassays, both blank and exposed yeast cell suspensions were placed into open-topped cylindrical measurement glass vessels (volume 25 mL) at controlled temperature (25.0 ± 0.1 °C) and under constant magnet stirring (200 rpm). During the 24 h exposures, all the measurement glass vessels were covered with 55 mm diameter cellulose filter discs (Whatman^®^ qualitative filter paper, Grade 1; Sigma Aldrich, Milan, Italy) to limit water evaporation (<5%) while ensuring a proper O_2_ exchange between air and yeast cell suspensions. The non-proliferation condition is a key aspect of the respirometric bioassay [36]. To guarantee a non-proliferation condition for the yeast *S. cerevisiae*, throughout the 24 h exposures, both blank yeast cell suspensions group and exposed yeast cell suspensions group underwent the same nutrient starving condition. For each blank and exposed yeast cell suspension, the non-proliferation condition was verified by comparing the results of optical density measurements at the wavelength of 525 nm (OD_525nm_) performed at the beginning of each exposure and after each respirometric bioassay (ΔOD_525nm_). The OD_525nm_ measurements were performed in quartz cuvettes with 1 cm optical path lengths against high-purity deionized water as reference (UV2 UV/Visible, Unicam Instruments, England). For all respirometric bioassays performed, no significant cellular proliferation of *S. cerevisiae* was observed (ΔOD_525nm_ ≤ 0.005 AU). Respirometric bioassays were performed in an open system (i.e., dissolved oxygen into the yeast cell suspensions was in equilibrium with the atmospheric oxygen) at controlled temperature (25.0 ± 0.1 °C) and under constant magnet stirring (200 rpm). At the end of 24 h exposure, each Clark-type oximeter were inserted in the eight measuring glass vessels for the simultaneous measurement and registration of the dissolved O_2_ concentration variations (expressed as Δppm) in the exposed and control yeast cell suspensions throughout the respirometric bioassays (see example in Figure 3). Aliquots of 2.50 mL of 3 mol/L glucose aqueous solution was added to each measuring glass vessel. As a direct consequence of the sudden subsequent change of cellular nutritional status, the yeast aerobic metabolism increased from the basal level to the maximum physiological value, and the dissolved O_2_ suddenly decreased (Figure 3). When the cellular consumption rate of the dissolved O_2_ becomes equal to the dissolving rate of atmospheric O_2_, the signals were reached a plateau (steady-state I; signal fluctuation ≤ 0.05 ppm) (Figure 3). At the steady state, 0.10 mL of 0.02 M NaN_3_ aqueous solution was added to each measuring glass vessel: NaN_3_ blocks the cellular O_2_ consumption through the inhibition mitochondrial Complex IV, which reduced O_2_ to H_2_O. The interruption of O_2_ cellular consumption triggered a progressively increase in dissolved O_2_ up to a second plateau (steady-state II; signal fluctuation ≤ 0.05 ppm) (Figure 3).

The analytical parameter considered in the respirometric bioassay was the variation of the dissolved O_2_ (Δppm O_2_) between the two steady-states. The percentage interference on cellular aerobic respiration (%ρ) is expressed as (Equation (1)):

%ρ = [1 − (Δppm O_2 exp_/Δppm O_2 blk_)] × 100 (1)

where ΔppmO_2 exp_ = mean of variations of the dissolved O_2_ (in ppm) for exposed yeast cell suspensions, and ΔppmO_2 blk_ = mean of variations of the dissolved O_2_ (in ppm) for control yeast cell suspensions.

In the described experimental conditions, %ρ values are readable as follows:

- %ρ < 0: hyperstimulation of cellular respiration in exposed yeast cell suspensions is over the maximum physiological rate (compared to control yeast cell suspensions);
- %ρ = 0: no effects on cellular respiration in exposed yeast cell suspensions;
- 0 < %ρ < 100: inhibition of cellular respiration in exposed yeast cell suspensions.

Considering that both blank yeast cell suspensions group and exposed yeast cell suspensions group underwent the same nutrient starving condition, any possible cellular process that could occur under this condition (e.g., induction of mitochondrial biogenesis and/or increase of mitochondrial mass) would occur in both groups. Thus, any variation of oxygen consumption between the exposed yeast cell suspensions group and blank yeast cell suspensions group can, therefore, only be associated with interference on cellular aerobic respiration induced by the exposure to tested chemical. To assess the repeatability of the measurements, four runs of the array measurements were performed on the same day, whereas reproducibility was verified on three different days, starting from freshly prepared solutions and yeast cell suspensions.

## 5. Conclusions

Cellular aerobic respiration of *S. cerevisiae* has proved to be a valid short-term in vitro toxicological endpoint to highlight mitochondrial interferences of BPA and BPB. Our results indicate that BPB possesses a similar hyperstimulation effect on *S. cerevisiae* cellular aerobic respiration and a 2-fold higher cellular respiration inhibition compared with BPA at the same dose. Findings from the respirometric bioassays after 24 h independent exposure to BPA and BPB allowed us to hypothesize, for the first time, a dose-dependent mode of action of two mechanisms of mitochondrial interferences (uncoupling of oxidative phosphorylation and oxidative stress). Future studies will investigate the time-dependence of the resultant effect of mitochondrial interference observed for 2,4-DNP also for BPA e BPB.

## References

[1] Kuruto-Niwa R., Tateoka Y., Usuki Y., Nozawa R. (2007). Measurement of bisphenol A concentrations in human colostrum. Chemosphere.

[2] Padmanabhan V., Siefert K., Ransom S., Johnson T., Pinkerton J., Anderson L., Tao L., Kannan K. (2008). Maternal bisphenol-A levels at delivery: A looming problem?. J. Perinatol..

[3] Caserta D., Ciardo F., Bordi G., Guerranti C., Fanello E., Perra G., Borghini F., La Rocca C., Tait S., Bergamasco B. (2013). Correlation of endocrine disrupting chemicals serum levels and white blood cells gene expression of nuclear receptors in a population of infertile women. Int. J. Endocrinol..

[4] Stahlhut R.W., Welshons W.V., Swan S.H. (2009). Bisphenol A data in NHANES suggest longer than expected half-life, substantial nonfood exposure, or both. Environ. Health Perspect..

[5] Balakrishnan B., Henare K.L., Thorstensen E.B., Ponnampalam A.P., Mitchell M.D. (2010). Transfer of bisphenol A across the human placenta. Am. J. Obstet. Gynecol..

[6] Mendonca K., Hauser R., Calafat A.M., Arbuckle T.E., Duty S.M. (2014). Bisphenol A concentrations in maternal breast milk and infant urine. Int. Arch. Occup. Environ. Health.

[7] Frazzoli C., Petrini C., Mantovani A. (2009). Sustainable development and next generation’s health: A long-term perspective about the consequences of today’s activities for food safety. Annali dell’Istituto Superiore di Sanità.

[8] Materials E.E.P.O.F.C. (2015). Scientific opinion on the risks to public health related to the presence of bisphenol A (BPA) in foodstuffs. EFSA J..

[9] Von Goetz N., Wormuth M., Scheringer M., Hungerbühler K. (2010). Bisphenol A: How the most relevant exposure sources contribute to total consumer exposure. Risk Anal..

[10] Pouokam G.B., Ajaezi G.C., Mantovani A., Orisakwe O.E., Frazzoli C. (2014). Use of Bisphenol A-containing baby bottles in Cameroon and Nigeria and possible risk management and mitigation measures: Community as milestone for prevention. Sci. Total. Environ..

[11] Ranjit N., Siefert K., Padmanabhan V. (2009). Bisphenol-A and disparities in birth outcomes: A review and directions for future research. J. Perinatol..

[12] Almeida S., Raposo A., Almeida-González M., Carrascosa C. (2018). Bisphenol A: Food exposure and impact on human health. Compr. Rev. Food Sci. Food Saf..

[13] Mercogliano R., Santonicola S. (2018). Investigation on bisphenol A levels in human milk and dairy supply chain: A review. Food Chem. Toxicol..

[14] Cunha S., Almeida C., Mendes E., Fernandes J. (2011). Simultaneous determination of bisphenol A and bisphenol B in beverages and powdered infant formula by dispersive liquid–liquid micro-extraction and heart-cutting multidimensional gas chromatography-mass spectrometry. Food Addit. Contam. Part A.

[15] Serra H., Beausoleil C., Habert R., Minier C., Picard-Hagen N., Michel C. (2019). Evidence for Bisphenol B endocrine properties: Scientific and regulatory perspectives. Environ. Health Perspect..

[16] Ike M., Chen M.Y., Danzl E., Sei K., Fujita M. (2006). Biodegradation of a variety of bisphenols under aerobic and anaerobic conditions. Water Sci. Technol..

[17] Grumetto L., Montesano D., Seccia S., Albrizio S., Barbato F. (2008). Determination of Bisphenol A and Bisphenol B residues in canned peeled tomatoes by reversed-phase liquid chromatography. J. Agric. Food Chem..

[18] Cunha S.C., Cunha C., Ferreira A.R., Fernandes J.O. (2012). Determination of bisphenol A and bisphenol B in canned seafood combining QuEChERS extraction with dispersive liquid–liquid microextraction followed by gas chromatography–mass spectrometry. Anal. Bioanal. Chem..

[19] Grumetto L., Gennari O., Montesano D., Ferracane R., Ritieni A., Albrizio S., Barbato F. (2013). Determination of five bisphenols in commercial milk samples by liquid chromatography coupled to fluorescence detection. J. Food Prot..

[20] Cobellis L., Colacurci N., Trabucco E., Carpentiero C., Grumetto L. (2009). Measurement of bisphenol A and bisphenol B levels in human blood sera from healthy and endometriotic women. Biomed. Chromatogr..

[21] Cunha S., Fernandes J.O. (2010). Quantification of free and total bisphenol A and bisphenol B in human urine by dispersive liquid-liquid microextraction (DLLME) and heart-cutting multidimensional gas chromatography-mass spectrometry (MD–GC/MS). Talanta.

[22] Liao C., Liu F., Guo Y., Moon H.-B., Nakata H., Wu Q., Kannan K. (2012). Occurrence of eight bisphenol analogues in indoor dust from the united states and several asian countries: Implications for human exposure. Environ. Sci. Technol..

[23] Yoshihara S., Mizutare T., Makishima M., Suzuki N., Fujimoto N., Igarashi K., Ohta S. (2004). Potent estrogenic metabolites of bisphenol A and bisphenol B formed by rat liver S9 fraction: Their structures and estrogenic potency. Toxicol. Sci..

[24] Kitamura S., Suzuki T., Sanoh S., Kohta R., Jinno N., Sugihara K., Yoshihara S., Fujimoto N., Watanabe H., Ohta S. (2005). Comparative study of the endocrine-disrupting activity of bisphenol A and 19 related compounds. Toxicol. Sci..

[25] Rosenmai A.K., Dybdahl M., Pedersen M., Van Vugt-Lussenburg B.M.A., Wedebye E.B., Taxvig C., Vinggaard A.M. (2014). Are structural analogues to bisphenol A safe alternatives?. Toxicol. Sci..

[26] Ng H.W., Shu M., Luo H., Ye H., Ge W., Perkins R., Tong W., Hong H. (2015). Estrogenic activity data extraction and in silico prediction show the endocrine disruption potential of bisphenol A replacement compounds. Chem. Res. Toxicol..

[27] Ullah A., Pirzada M., Jahan S., Ullah H., Turi N., Ullah W., Siddiqui M.F., Zakria M., Lodhi K.Z., Khan M.M. (2018). Impact of low-dose chronic exposure to bisphenol A and its analogue bisphenol B, bisphenol F and bisphenol S on hypothalamo-pituitary-testicular activities in adult rats: A focus on the possible hormonal mode of action. Food Chem. Toxicol..

[28] Barrera G., Gentile F., Pizzimenti S., Canuto R.A., Daga M., Arcaro A., Cetrangolo G.P., Lepore A., Ferretti C., Dianzani C. (2016). Mitochondrial dysfunction in cancer and neurodegenerative diseases: Spotlight on fatty acid oxidation and lipoperoxidation products. Antioxidants.

[29] Nakagawa Y., Tayama S. (2000). Metabolism and cytotoxicity of bisphenol A and other bisphenols in isolated rat hepatocytes. Arch. Toxicol..

[30] Ooe H., Taira T., Iguchi-Ariga S.M.M., Ariga H. (2005). Induction of reactive oxygen species by bisphenol A and abrogation of bisphenol A-induced cell injury by DJ-1. Toxicol. Sci..

[31] Xiao C., Wang L., Hu D., Zhou Q., Huang X. (2019). Effects of exogenous bisphenol A on the function of mitochondria in root cells of soybean (Glycine max L.) seedlings. Chemosphere.

[32] Gualtieri A.F., Iwachow M.A., Venara M., Rey R.A., Schteingart H.F. (2010). Bisphenol A effect on glutathione synthesis and recycling in testicular Sertoli cells. J. Endocrinol. Investig..

[33] Lukacova J., Jambor T., Knazicka Z., Tvrda E., Kolesarova A., Lukac N. (2015). Dose- and time-dependent effects of bisphenol A on bovine spermatozoa in vitro. J. Environ. Sci. Health Part A.

[34] Ikhlas S., Usman A., Ahmad M. (2019). In vitro study to evaluate the cytotoxicity of BPA analogues based on their oxidative and genotoxic potential using human peripheral blood cells. Toxicol. Vitr..

[35] Dragone R., Cheng R., Grasso G., Frazzoli C. (2015). Diuron in water: Functional toxicity and intracellular detoxification patterns of active concentrations assayed in tandem by a yeast-based probe. Int. J. Environ. Res. Public Health.

[36] Grasso G., Caracciolo L., Cocco G., Frazzoli C., Dragone R. (2018). Towards simazine monitoring in agro-zootechnical productions: A yeast cell bioprobe for real samples screening. Biosensors.

[37] Dragone R., Frazzoli C., Grasso G., Rossi G. (2014). Sensor with intact or modified yeast cells as rapid device for toxicological test of chemicals. J. Agric. Chem. Environ..

[38] Dragone R., Grasso G., Frazzoli C., Asongalem A.E., Orisakwe O.E. (2012). Biosensoristic devices: Monitoring and diagnostics in agro-zootechnical productions. Cameroon-Nigeria-Italy Scientific Cooperation: Veterinary Public Health and Sustainable Food Safety to Promote “One Health/One Prevention”.

[39] Frazzoli C., Dragone R., Mantovani A., Massimi C., Campanella L. (2007). Functional toxicity and tolerance patterns of bioavailable Pd(II), Pt(II), and Rh(III) on suspended Saccharomyces cerevisiae cells assayed in tandem by a respirometric biosensor. Anal. Bioanal. Chem..

[40] Dragone R., Grasso G., Muccini M., Toffanin S. (2017). Portable bio/chemosensoristic devices: Innovative systems for environmental health and food safety diagnostics. Front. Public Health.

[41] Dragone R., Ermilov L., Grasso G., Maggioni S., Mantovani A., Frazzoli C. (2016). Antioxidant power as biochemical endpoint in bread for screening and early managing quality and toxicant-related safety anomalies in food production. Food Chem. Toxicol..

[42] Skulachev V.P. (1998). Uncoupling: New approaches to an old problem of bioenergetics. Biochim. et Biophys. Acta (BBA) Bioenerg..

[43] Moses V., Smith M.J.H. (1960). Uncoupling reagents and metabolism. 2. Effects of 2:4-dinitrophenol and salicylate on glucose metabolism in baker’s yeast. Biochem. J..

[44] Bereketoglu C., Arga K.Y., Eraslan S., Mertoglu B. (2016). Analysis of transcriptional profiles of Saccharomyces cerevisiae exposed to bisphenol A. Curr. Genet..

[45] Brand M. (2000). Uncoupling to survive? The role of mitochondrial inefficiency in ageing. Exp. Gerontol..

[46] Kadenbach B. (2003). Intrinsic and extrinsic uncoupling of oxidative phosphorylation. Biochim. et Biophys. Acta (BBA) Bioenerg..

[47] Brookes P.S. (2005). Mitochondrial H^+^ leak and ROS generation: An odd couple. Free. Radic. Biol. Med..

[48] Aon M., Cortassa S., O’Rourke B. (2010). Redox-optimized ROS balance: A unifying hypothesis. Biochim. et Biophys. Acta (BBA) Bioenerg..

[49] Bakker B.M., Overkamp K.M., Van Maris A.J., Kötter P., Luttik M.A., Van Dijken J.P., Pronk J.T. (2001). Stoichiometry and compartmentation of NADH metabolism in Saccharomyces cerevisiae. FEMS Microbiol. Rev..

[50] Balaz S. (2009). Modeling kinetics of subcellular disposition of chemicals. Chem. Rev..

[51] Okamura E., Kakitsubo R., Nakahara M. (1999). NMR Determination of the delivery site of bisphenol A in phospholipid bilayer membranes. Langmuir.

[52] Okamura E., Wakai C., Matubayasi N., Sugiura Y., Nakahara M. (2004). Limited slowdown of endocrine-disruptor diffusion in confined fluid lipid membranes. Phys. Rev. Lett..

[53] Broniatowski M., Sobolewska K., Flasiński M., Wydro P. (2016). Studies on the interactions of bisphenols with anionic phospholipids of decomposer membranes in model systems. Biochim. et Biophys. Acta (BBA) Biomembr..

[54] Janssen M., Koorengevel M., De Kruijff B., De Kroon A. (1999). Transbilayer movement of phosphatidylcholine in the mitochondrial outer membrane of Saccharomyces cerevisiae is rapid and bidirectional. Biochim. et Biophys. Acta (BBA) Biomembr..

[55] Joshi A.S., Zhou J., Gohil V.M., Chen S., Greenberg M.L. (2009). Cellular functions of cardiolipin in yeast. Biochim. et Biophys. Acta (BBA) Bioenerg..

[56] Koshkin V., Greenberg M.L. (2002). Cardiolipin prevents rate-dependent uncoupling and provides osmotic stability in yeast mitochondria. Biochem. J..

[57] Paradies G., Paradies V., De Benedictis V., Ruggiero F.M., Petrosillo G. (2014). Functional role of cardiolipin in mitochondrial bioenergetics. Biochim. et Biophys. Acta (BBA) Bioenerg..

